# Supplementary material for: Deletion of a kinesin I motor unmasks a mechanism of homeostatic branching control by neurotrophin-3
Source: eLife. 2015 Jun 15;4:e05061. doi: 10.7554/eLife.05061 (PMC4467164; doi:10.7554/eLife.05061)
Supplement: Supplementary file 1. — Description of zebrafish mutant and transgenic lines used in this study. DOI: http://dx.doi.org/10.7554/eLife.05061.026 [file elife05061s001.docx]

**Supplementary File 1:** Description of zebrafish mutant and transgenic lines used in this study.

| **Transgenic or mutant zebrafish line used in this study** | **Gene affected** | **Description** | **Original Reference** |
| --- | --- | --- | --- |
| *kif5aa*^*162^ | *kif5aa* | TALEN mediated loss-of-function allele of *kif5aa.* | generated in this study |
| *vertigo (ver)* | *kif5aa* | Mutant strain identified in an ENU based genetic screen showing the same Retinal Ganglion Cell (RGC) axon delayed growth phenotype as *kif5aa*^*162^ and not complementing with this allele. | ([Xiao et al., 2005](#_ENREF_103)) |
| *lakritz (lak)* | *atoh7* | Loss-of-function allele from an ENU based genetic screen. *Lak* mutant embryos fail to specify RGCs and lack therefore functional connections between the retina and other brain areas. | ([Kay et al., 2001](#_ENREF_42)) |
| *blumenkohl (blu)* | *vglut2a* | Loss-of-function allele from an ENU based genetic screen. Loss of *vglut2a* leads to reduced synaptic transmission between RGCs and postsynaptic tectal neurons. Furthermore *blu* mutants grower larger RGC axonal arbors. | ([Smear et al., 2007](#_ENREF_84)) |
| *Tg(BGUG*) also described as *PGUG (Pou4f3:Gal4,UAS:mGFP*) |  | Brn3C:Gal4,UAS:mGFP transgenic line that labels between one and ten RGCs per retina projecting mainly to the SFGS layers of the optic tectum with membrane localized GFP. SFGS = stratum fibrosum et griseum superficiale. | ([Xiao and Baier, 2007](#_ENREF_102)) |
| *Tg(Pou3f4:Gal4)* |  | A promoter fragment of the *pou3f4* (also know as *brn3C*) gene drives expression of the transactivator Gal4 in a subpopulation of RGCs projecting to the SO and SFGS layers of the optic tectum. | ([Xiao and Baier, 2007](#_ENREF_102)) |
| *Tg(Isl2b:Gal4)* |  | A promoter fragment of the *isl2b* gene drives expression of the transactivator Gal4 in all RGCs of the retina. | ([Ben Fredj et al., 2010](#_ENREF_11)) |
| *Tg(HuC:GCaMP5G)* |  | A promoter fragment of the *huC* gene (also known as *elavl3*) drives expression of the genetically encoded calcium indicator GCaMP5G in almost all differentiated neurons. | ([Ahrens et al., 2013](#_ENREF_3)) |
| *Tg(UAS:GCaMP3)* |  | The expression of the genetically encoded calcium indicator GCaMP3 is dependent of the activation of a 5’ upstream activator sequence (UAS). | ([Warp et al., 2012](#_ENREF_96)) |
| *Tg(gSA2AzGFF49A)* |  | Gene trap line with an insertion of a GFF (optimized Gal4) transactivator into the zebrafish genome. GFF is expressed from 2dpf onwards in tectal periventricular neurons. | ([Muto et al., 2013](#_ENREF_59)) |
| *Tg(Pou4f3:mGFP)* |  | A promoter fragment of the *pou3f4* (also know as *brn3C*) gene drives expression of membrane bound GFP in a subpopulation of RGCs projecting to the SO and SFGS layers of the optic tectum. | ([Xiao et al., 2005](#_ENREF_103)) |
| *Tg(Shh:eGFP)* |  | A promoter fragment of the *shh* gene drives expression of eGFP in developing neurons in the retina starting with RGCs. | ([Neumann and Nuesslein-Volhard, 2000](#_ENREF_62)) |
| *Tg(UAS:RFP, cry:eGFP)* |  | The expression of a red fluorescent protein (RFP) is dependent of the activation of a 5’ upstream activator sequence (UAS). A crystalline promoter fragment (cry) induces expression of eGFP in the lens as transgenesis reporter. | ([Auer et al., 2014](#_ENREF_8)) |
| *Tg(UAS:SypGFP)* |  | The expression of a synpatophysin-GFP fusion protein (SypGFP) is dependent of the activation of a 5’ upstream activator sequence (UAS). SypGFP marks presynaptic clusters and synaptic vesicles. | ([Meyer and Smith, 2006](#_ENREF_54)) |
| *Tg(UAS:BoTxLCB-GFP)* |  | The expression of Botulinum toxin light chain B is dependent of the activation of a 5’ upstream activator sequence (UAS). The Toxin leads to loss of synaptic transmission by inhibition of synaptic vesicle fusion. | generated in this study |
