# Supplementary material for: Deletion of a kinesin I motor unmasks a mechanism of homeostatic branching control by neurotrophin-3
Source: eLife. 2015 Jun 15;4:e05061. doi: 10.7554/eLife.05061 (PMC4467164; doi:10.7554/eLife.05061)
Supplement: Supplementary file 2. — Primers used in this study. DOI: http://dx.doi.org/10.7554/eLife.05061.027 [file elife05061s002.docx]

**Supplementary File 2:** Primers used in this study.

| **Primer** | **Sequence (5’ to 3’)** | **Comment** |
| --- | --- | --- |
| tagRFPCaax_rev | GATCGCGGCCGCTCAGGAGAGCACACACTTGCAGCTCATGCAGCCGGGGCCACTCTCATCAGGAGGGTTCAGCTTATTAAGTTTGTGCCC | To amplify tagRFPCaax |
| tagRFP_fwd | CCCGGGATCCACATGGTGTCTAAGGGCGAAG | To amplify tagRFPCaax |
| ntf3_fwd | GCTTAAGCTTGACCATGTCCATCTTGCTGTACG | To amplify a ntf3 full length-E2A fusion |
| E2A-GGG-ntf3_rev | CTCCAGCCAATTTCAAGAGAGCATAATTAGTACACTGGCCGCCGCCCGTCCTTCTATGTTTCTTTGAGAGC | To amplify a ntf3 full length-E2A fusion |
| E2A-GGG-tagRFP_fwd | CTTGAAATTGGCTGGAGATGTTGAGAGCAACCCAGGTCCCGGCGGCGGCGTGTCTAAGGGCGAAGAGCTG | To amplify a E2A-tagRFP fragment |
| tagRFP_rev | AGTCGCGGCCGCTCAATTAAGTTTGTGCCC | To amplify a E2A-tagRFP fragment |
| drntrk3a_fwd | GCTTAAGCTTACCATGGATTTATTCTCCATCCCGCC | To amplify a dominant negative ntrk3A fragment |
| drntrk3a_rev | CCTCGCCCTTGCTCACTCCTCCTCCATAAGTAGCAGGCTTGTTACAGTTGTGCCC | To amplify a dominant negative ntrk3A fragment |
| GGG-eGFP_fwd | GGAGGAGGAGTGAGCAAGGGCGAGGAGCTGTTCACCG | To amplify a triple glycine eGFP fragment |
| eGFP_rev | AGTCGCGGCCGCTCACTTGTACAGCTCGTCCATGCCG | To amplify a triple glycine eGFP fragment |
| ntf3_fwd | GTACCTTTATGGTATCTCCGCC | To generate an *in-situ* probe |
| ntf3_rev | CATTTTTCACGTCCTTCTATGTTTC | To generate an *in-situ* probe |
| ntrk3a_fwd | GATGCTGGTGGCTGTCAAGACC | To generate an *in-situ* probe |
| ntrk3a_rev | CTAGCCCAGGATATCCAGGTAGAC | To generate an *in-situ* probe |
| ntrk3b_fwd | GACCATGGATGTATGGTTGTGTTC | To generate an *in-situ* probe |
| ntrk3b_rev | TTACAGTTCGACCAGCTGAAGGTGC | To generate an *in-situ* probe |
| kif5aa_fwd | AGCATCGTCTACTCGACGGGGTTTT | To generate an *in-situ* probe |
| kif5aa_rev | GCTGCTCCCGTCTTACTGACCTTCT | To generate an *in-situ* probe |
| kif5aa_qpcr_f | CGGCACCATCTTTGCCTATG | For qRT-PCR |
| kif5aa_qpcr_r | ATCTGCTGCGGGTCGTGAA | For qRT-PCR |
| ntf3_qpcr_f | TTACCTTCATGTCGGCTCTGCTG | For qRT-PCR |
| ntf3_qpcr_r | CGCGAGGAACATCACGTACAG | For qRT-PCR |
| bdnf_qPCR_f | CTTGAGGTGGAAGGGGAAGCG | For qRT-PCR |
| bdnf_qPCR_r | GTAACGGCGGCTCCAAAGGC | For qRT-PCR |
| ntf4_qpcr_f | CACGGAGGTGACAAAGAGGC | For qRT-PCR |
| ntf4_qpcr_r | GGCGGGCTCTAGGAACGTG | For qRT-PCR |
| ntf7_qpcr_f | AGCCTGCATTGGACTCTGGC | For qRT-PCR |
| ntf7_qpcr_r | CAGGATGAGCAGGACCAGCG | For qRT-PCR |
| ngf_qpcr_f | AGATGCCACGCTGGTCGATAC | For qRT-PCR |
| ngf_qpcr_r | ACCACCGCATGGGCTCAAC | For qRT-PCR |
| EF1alpha_qpcr_f | CTGGAGGCCAGCTCAAACAT | For qRT-PCR |
| EF1alpha_qpcr_r | ATCAAGAAGAGTAGTACCGCTAGCATTAC | For qRT-PCR |
| RPL13alpha_qpcr_f | TCTGGAGGACTGTAAGAGGTATGC | For qRT-PCR |
| RPL13alpha_qpcr_r | AGACGCACAATCTTGAGAGCAG | For qRT-PCR |
