# Supplementary material for: Deletion of a kinesin I motor unmasks a mechanism of homeostatic branching control by neurotrophin-3
Source: eLife. 2015 Jun 15;4:e05061. doi: 10.7554/eLife.05061 (PMC4467164; doi:10.7554/eLife.05061)
Supplement: Source code 1. — Source code for moving bar stimulus. DOI: http://dx.doi.org/10.7554/eLife.05061.028 [file elife05061s003.docx]

**Source Code 1** Source Code for moving bar stimulus

% Generating an x-moving bar.

% rate = plus (towards right) or minus (towards left)

% initPos = initial position with +1 farthest right and -1 farthest left

% interval = time in s between end of one run and new reset of the bar at the beginning

function Rect_Move(rate, initPos, interval, spotRadius, angle)

rotationRadius = 400;

% The radius of the screen along x-axis of the screen in pixel.

try

KbName('UnifyKeyNames');

Screen('Preference', 'SkipSyncTests', 1);

% Removes the blue screen flash and minimize extraneous warnings.

Screen('Preference', 'VisualDebugLevel', 0);

oldEnableFlag = Screen('Preference', 'SuppressAllWarnings', 1);

% Find out how many screens and use largest screen number.

whichScreen = max(Screen('Screens'));

% Open a new window.

[ window, windowRect ] = Screen('OpenWindow', whichScreen);

% Set colors.

white = WhiteIndex(window);

% parameters

spotDiameter = spotRadius * 64;

spotRect = [0 0 spotDiameter 100*spotDiameter];

centeredspotRect = CenterRect(spotRect, windowRect); % Center the spot.

scalePos = initPos;

% Get the coordinates of the center of the screen to use for rotation

ScRect = Screen('Rect',window);

centX = ScRect(3)/2;

centY = ScRect(4)/2;

% Set up the timer.

startTime = now;

ran = false;

% Loop until key is pressed.

while true

numberOfSecondsElapsed = (now - startTime) * 3600 * 24;

if ~ran

done = false;

while ~done

xOffset = rotationRadius * scalePos;

yOffset = 0;

offsetCenteredspotRect = OffsetRect(centeredspotRect, xOffset, yOffset);

%rotate rects

Screen('glPushMatrix', window)

Screen('glTranslate', window, centX, centY)

Screen('glRotate', window, double(angle), double(0),double(0));

Screen('glTranslate', window, -centX, -centY)

Screen('FillRect', window, [0 0 0], offsetCenteredspotRect);

Screen('glPopMatrix', window)

Screen('Flip', window);

[ keyIsDown ] = KbCheck;

scalePos = scalePos + rate;

if abs(scalePos) > 1

done = true;

end

if keyIsDown

break;

end

end

ran = true;

end

Screen(window, 'FillRect', white); % set whole screen white

Screen('Flip', window);

if numberOfSecondsElapsed >= interval

ran = false;

startTime = now;

scalePos = initPos;

end

if keyIsDown

break;

end

[keyIsDown] = KbCheck;

end

Screen('CloseAll');

catch

Screen('CloseAll');

psychrethrow(psychlasterror);

Screen('Preference','SuppressAllWarnings', oldEnableFlag);

end

return
